# Physiological Chemistry

**Published:** 1854-02

**Authors:** Benjamin Silliman

**Affiliations:** Professor of Medical Chemistry and Toxicology in the University of Louisville


### Art. V.—PHYSIOLOGICAL CHEMISTRY.

BY BENJAMIN SILLIMAN, JR., M.A., M.D., PROFESSOR OF MEDICAL CHEMISTRY AND TOXICOLOGY IN THE UNIVERSITY OF LOUISVILLE.

It is not our purpose to attempt a full review of the elaborate and truly learned works upon Physiological Chemistry whose titles are given below. * Such a review would, if sufficiently full to cover the whole ground, extend to an unreasonable length, and embrace details not interesting or even generally understood by the body of the medical profession.

Simon's Chemistry of Man, which appeared in 1842, marked an era in chemical physiology which has been followed and sustained by immense activity in researches bearing upon the chemistry of life. The results of the zeal and industry of the army of workers, which flocked to the laboratories of Paris, Giessen,

* Physiological Chemistry, by Professor C. G. Lehmann, translated from the second (German) edition, by Geo. E. Day, M. D., F. R. S., Fellow of the Royal College of Physicians, and Professor of Medicine in the University of St. Andrews. London: 1851, 1853. Printed for the Cavendish Society. Vols. I, II; 8vo., pp. 455 and 465, respectively

Atlas of Physiological Chemistry, (consisting of microscopic figures,) being a supplement to Lehmann's Physiological Chemistry, by Dr. Otto Funke, of the University of Leipsic. London: 1853; printed for the Cavendish Society; quarto, fifteen plates, (with ninety figures,) engraved on stone and printed in colors, with explanatory text by the author, quarto, pp. 29.

Robin et Verdeil Traite de Chimie Anatomique et Physiologique, Normale et Pathologique, &c. Treatise on Anatomical and Physiological Chemistry, Normal and Pathological; or, the proximate principles, both morbid and normal, which compose the bodies of man and mammiferous animals, by Chas. Robin and F. Verdeil; with an Atlas of forty-five plates, in part colored. 3 vols., 8vo., pp. 728, 584, and 595. Paris: J. B. Bailliere, 1853.

Lehrbuch der Zoochemie, von H. W. Heintz. Berlin: 1853; 1 vol., 8vo., pp. 1107, with two plates.

Utrecht, and Berlin, to follow up or refute the doctrines of Liebig, so eloquently announced in his well-known works,—these results, so long appearing as an intricate and unconnected mass of facts in organic chemistry, have now assumed a degree of systematic order and compact connection, worthy of the labor which they have cost.

We shall in the present article confine ourselves almost exclusively to the work of Lehmann, trusting on a future occasion to resume the consideration of the others. Lehmann's work has been translated and published for the members of the Cavendish Society.

The Cavendish Society is one of those organizations, established during the past few years in England, whose object is to publish, solely for subscribers, rare and important works, either historical or contemporaneous, which no publisher can be found willing to undertake. The Sydenham Society has thus conferred lasting benefits upon medical literature, by its now long catalogue of rare and valuable treatises. The Cavendish is doing the same service for the chemical reader. One of the most important benefits conferred by these associations is the translation, by competent hands, of the current medical literature of Germany;—researches published in German only, are a sealed record to the greater number of even well educated readers of English, and yet we know that the most laborious workers are Germans, and certainly some of the most original and important observations in all departments of science are made in Germany. The Cavendish Society employed Dr. Geo. E. Day, F. R. S., to translate "Lehmann's *Lehrbuch der Physiologischen Chemie.*" Dr. Day's fitness for this task is evinced both by the ability with which he has rendered the original, and by his experience as the translator of Simon's Chemistry.

Two only of the three volumes of Lehmann's work have appeared in English, and the third were issued in Germany only toward the close of last year. It is accompanied by Dr. Funke's Atlas of Physiological Chemistry, a distinct work, but prepared with special reference to the elucidation of Lehmann's Chemistry. The quarto plates of this work, fifteen in number, embrace ninety separate microscopic fields, exhibiting everything which can be seen in form, structure, or color, illustrative of the various morphological elements and organic substrata of the animal organism, both in health and disease, as far as they have been observed by the author. Every detail is original and drawn from the microscope, and nothing is copied from other observers.

An apology for chemistry as the younger sister of medicine and claiming to be her teacher, is no longer demanded of the physiological chemist, and in the pendulation of professional opinion, the votary of this science is led almost to fear that the high expectations of the future development of a rational system of therapeutics, diagnosis and practice, as a result of zoochemical researches, may be met with disappointment. Not that he fears, or would limit the resources of his science, under the guidance of a rigorous induction from facts of undoubted authenticity; but, rather, as LEHMANN remarks, that "enthusiasm in the cause of organic chemistry has degenerated amongst many physiologists and physicians into fanaticism, which, even in the best cause, tends to invalidate a host of truths in its endeavors to uphold some single fact." We might be disposed to ask whether its most zealous partizans have not retarded, rather than accelerated, the period at which it will attain its proper share of appreciation, and its just recognition. In commencing, therefore, the study of physiological chemistry, nothing is more important than clearly to understand the nature of the results which this department of science is now capable of yielding, and the requirements which, in its present state of development, it fulfils; and to ascertain the cause, the means, and the methods, most likely to lead us safely within its domain, and at the same time the best adapted to promote its further progress.

The subject of physiological chemistry naturally divides itself into three grand divisions—each capable of separate investigation, but following each other in a determinate logical sequence. These are, first, what are called the "organic substrata of the animal organism; i. e., such substances as are usually intended by the term "proximate principles," (and which is a translation of the French term *principes immédiats.*) Such are the various organic acids, the alkaloids, the salts of fatty acids (fats), sugar, coloring matters, protein compounds, (albumin, fibrin, etc.), and the various mineral constituents of the body. It is to the history of these, as the corner stone of all rational knowledge of animal chemistry, that our author devotes his first volume.

The second division includes the "animal juices," regarded as the variable results of the union and secretion of the members of the first division, under the influence of the various processes of life-chemistry, occurring in the animal organism, both in health and disease. The discussion of this important class of products—which occupies the position of an intermediate link between the theory of the organic substrata and that of the zoo-chemical processes—fills the second volume.

The third division of the subject is the study of the zoo-chemical processes, based on zoo-chemistry and the theory of the animal juices—the theory of the metamorphosis of tissues, of nutrition and secretion, of digestion and respiration. This is the sum and crown of the whole subject, and fills the third volume of LEHMANN, which has not yet appeared in English, but is now in process of preparation by the Cavendish Society. Meanwhile, let the reader of English content himself with a careful study of the two volumes already in his hands, convinced that he cannot too thoroughly master the subjects therein treated, so fundamental to any rational and practical deductions which are to be drawn from them toward forming a just notion of the metamorphosis of the tissues.

LEHMANN's work is opened by an introduction, occupying twenty-four pages, which the author calls a "Methodological Introduction," and in which he discusses in a most masterly manner, and in the spirit of a cautious philosophy, the sure principles which should guide the scientific enquirer in the prosecution of physiological chemistry. From this "Introduction" we propose to make some rather ample extracts, as it is not possible to put the important truths it contains—as the ripe results of long study and great genius and skill—in more forcible and compact expression. We commend to the attention of men who are vainly striving after a "true theory" for the explanation of vital phenomena, not as the result of legitimate investigation, but as the off-spring of ingenious presumption, the following:—

"It is only by the application of abstract physical laws, by the establishment of certain momenta of empirically observed phenomena, and by a steady adherence to safely guiding maxims,—in short, by logical sequence,—that we can advance in the investigation of vital phenomena. It would almost seem as if medicine, in the earlier periods of its history, had cast a shadow over those kindred sciences which are able to afford it aid and support, clouding even their brightest points. It has thus been found impracticable at once to rid medicine, notwithstanding its assumed physiological character, of the mania of attempting to explain everything by the old system of hypotheses; and hence this science has derived less benefit than many others from the exact method of physical enquiry, having simply borrowed certain materials from chemistry and the kindred natural sciences, and substituted, in the place of the older vagaries of natural philosophy, various chemical phrases and high sounding terms, scarcely less devoid of true import than the former. This deficiency in logical sequence, which we so frequently at present encounter in medicine, has unfortunately also infected animal chemistry; for here likewise facts have not been sufficiently distinguished from hypotheses, or hypothesis from fiction. This is more easily accounted for in physiological than in pure general chemistry; for while the latter treats almost exclusively of palpable phenomena and of well-established facts, which easily admit of being reduced to definite laws, in the former we must necessarily have recourse to experiments and natural investigations, whose success must in a great measure depend on individual operations of the mind. Zoo-chemical processes are the most complicated of any comprised in the domain of natural enquiry; but such processes are not capable of tangible demonstration, but must be divined, or rather, intellectually apprehended. Our senses are incapable of perceiving the causal connexion of things, or the logical succession of phenomena; thus we do not see motion, but simply recognize it by the result of the changes effected by it; we do not perceive heat, but simply the variations of the temperature, and the results to which they give rise, &c. Hence it is not our senses which here deceive us, but the judgment which we form regarding the objects presented to us by the perceptive faculties. The causal connexion of several allied phenomena, (*i. e.*, a process,) can therefore only be comprehended by the subjective combination of individual objects perceived by the senses, and not by sensuous intuition alone. But as soon as we subject to investigation the highly complicated chemical phenomena of life, we enter upon the actual domain of hypothesis. It unfortunately happens, however, that the correct logical conception of an hypothesis has been completely lost sight of, and its place supplied by the vaguest fictions; whence the term has fallen into such discredit that many have been desirous of setting aside all hypotheses, unmindful that even the simplest form of experiment cannot be prosecuted without their aid. Hypotheses are indispensable in every physical enquiry, and must constitute the base of every experiment, as they are in fact merely the subjection of our thoughts and mode of intuition to the reality of phenomena. The question, however, always is, whether the facts at our command logically justify such a procedure, since where such is not the case, the deduction at which we arrive is undeserving the name of an hypothesis, and is a mere fiction, supported at best on a hypothetical foundation.

Physiological chemistry has given rise to many delusions of this nature, owing to its imperfect development, and to the necessity presented by physiology and pathology for chemical elucidation. Some few isolated deductions were drawn from superficial chemical experiments, and arranged in a purely imaginary connexion by the aid of chemical symbols and formulæ, for whose establishment analysis in many cases did not even afford any sanction. Thus, for instance, in the attempt to form a conclusion regarding the metamorphosis of the blood from an elementary analysis of its solid residue and of the composition of the individual constituents of the excretions, there is an utter absence of all scientific groundwork; for, independently of the fact that the elementary analysis of so compound a matter as the blood is incapable of yielding any reliable results, and cannot, therefore, justify the adoption of any special chemical formulæ, it is assuredly most illogical to attempt to compare the composition of the blood collectively, with that of the separate excrementitious matters. In such deductions, expressed by chemical formulæ, the addition of atoms of oxygen, and the subtraction of those of water, carbonic acid, and ammonia are wholly arbitrary: for chemical analyses do not afford the slightest grounds for the majority of these equations. When, on the other hand, we have seen uric acid decomposed by different oxidising agents into urea and other bodies, and when, further, we find the quantity of uric acid increased in the urine in those cases where a diminished quantity of oxygen is proved to be contained in the blood, we are justified in concluding that also in the animal organism a portion, at least, of the urea found in the urine must have been produced by the oxidation of the uric acid. In the formulæ which expresses this deduction, we have an hypothesis, but a well-grounded one, which, although requiring further confirmation, is yet wholly different from the frequently condemned, but rarely avoided, abuse of chemical symbols. Chemical equations having no other foundation than the presumed infallibility of empirical formulæ, must, however, cause us to deviate from the path of physical enquiry, and involve us in a chaos of the most untenable delusions. Thus, for instance, a chemical equation might lead us to conclude that glycine (glycocoll) was the source of urea and lactic acid in the metamorphosis of the animal tissues; for we might conclude that two equivalents of hydrate of glycine were decomposed into the above-named substances according to the formula, $C_8 H_{10} N_2 O_8 = C_2 H_4 N_2 O_2 + C_6 H_5 O_5 . HO$. All experiments hitherto instituted with glycine are, nevertheless, opposed to such a disintegration. If, then, we would deduce urea and lactic acid from glycine, which has not been proved to exist in the blood, we should be neglecting the most comprehensive rule of logic, according to which one hypothesis cannot be supported by another. It has, however, unfortunately been too much the practice in recent times to employ far more complicated equations as supports for such purely subjective modes of contemplation, by which a semblance of the most exact method of investigation has been assumed. By these means a number of chemical fictions have supplanted the fancies of that speculative natural philosophy which in earlier times encumbered the study of physiology and pathology, and have plunged medicine into the midst of a new labyrinth of untenable theories."

The author then proceeds to indicate a further cause of the partial failure of the application of chemistry in vital phenomena, in the imperfect causal connexion among the different branches of natural science. He illustrates this by reference to the diagnosis afforded by pathological anatomy, which has received so little attention on the part of those who have conducted pathologico-chemical enquiries, while on the other hand the adherents of the pathologico-anatomical school have made full use of chemical phrases and fictions without an adequate acquaintance with the general science of chemistry. If chemical investigations, regarding objects belonging to pathological anatomy, would aspire to a scientific value, and if they are to afford any true elucidation of pathological processes, it will assuredly be admitted that the question should be adequately considered from an anatomical and diagnostic point of view. Yet every day presents us with instances of the most flagrant neglect of this self-evident proposition. How frequently we hear of the chemical examination of diseased bones, without any regard to a diagnosis at all in accordance with the present condition of pathological anatomy. We even more frequently meet with similar inconsistencies in the investigation of diseased animal fluids.

We cannot read the following bold and manly sentences without wishing that a similar courage in facing the truth could animate all teachers and investigators—so strongly contrasted with the too prevalent desire evident in many writers to slur over the weak points of their subjects with the semblance of fact or argument in hope of making a strong case.

"A third circumstance which has led to misconceptions in physiological chemistry depends upon an over-estimate of the value of chemical auxiliaries, and a complets ignorance of the present condition of organic chemistry. Have the numerous analyses of morbid blood, instituted during the last few years, fulfilled the expectations of physicians? With all due gratitude to the indefatigable investigators who, with no other aid than that which zoochemistry could offer, boldly attempted to throw light on those obscure enquiries, it must be admitted that, when we seriously enquire into the recompense of all their labours and sacrifices, we find that the result, although too dearly bought, was altogether inadequate to satisfy the requirements of pathology. Have the numerous analyses of urine led to much more than the assumption of several new species of disease, or so-called diatheses? Although we might have anticipated greater results, we can hardly wonder that the efforts hitherto made should either wholly or partially have deceived our expectations; for although these investigations may have rendered chemistry no unworthy auxiliary to a physical diagnosis, analyses of morbid products could hardly afford an insight into the chemical laboratory of the organism, while the means were wanting to prosecute them with the scientific accuracy attainable in the case of mineral analyses. Animal chemistry is still wholly unable to afford us a precise, and at the same time a practically useful method of investigating the blood; and how should it be otherwise while we continue to be in doubt regarding the chemical nature of its ordinary constituents? The mineral substances of normal blood are not yet determined, or, at all events continue to be made the subject of dispute; we scarcely know the names of the fatty matters it contains; one of its most important constituents, fibrin, cannot be chemically exhibited in a pure state; we are ignorant of the nature and mode of secretion of the globulin of the blood-corpuscles; we are still far from being able to separate and determine the so-called protein oxides; and we are also ignorant of the excrementitious substances occurring in the blood. How then, amidst these and a thousand other uncertainties and doubts, can an investigation of the blood be scientifically and trustworthily conducted? We analyse healthy and morbid milk, and yet we are ignorant of the substances whose admixture we have termed casein. The urine, in its morbid condition, presents many varieties; and yet our knowledge of this secretion, frequently as it has been analysed, amounts to little more than an acquaintance with the quantitative relations of some of its principal constituents; creatinine and hippuric acid have not been determined by any analysis, and doubts are still entertained by some chemists, (although most unjustly,) regarding the presence of the latter in human urine, while absolutely nothing is known regarding the most important pigment of this secretion. Many experiments have been made and theories broached on nutrition and digestion, and yet to almost the present day the existence of lactic acid in the gastric juice has been contested. Although hypotheses are not wanting regarding the mode of action of pepsin, we know nothing of its chemical nature, and we are wholly ignorant of the proximate metamorphosis of albuminous bodies in the stomach during the process of digestion. Will Mulder be able, even with his most accurate analyses, to support his protein theory by the aid of sulphamide and phosphamide? or is this term destined merely to indicate a past epoch of organic chemistry? When such is the state of animal chemistry, can we wonder that there should be obscurity regarding the chemical processes in the animal body, their various isolated and combined actions, their causal connexion and their dependence on external influences and internal conditions? Unfortunately, we might be led to believe, from the lectures and writings of many physicians, that, trusting to the aphoristic and often highly apodictic assertions of certain chemists they felt secure of having reached the object of their enquiries. Although at present little more than the direction is indicated we may hope in due time, and after innumerable efforts, to see our endeavors crowned with success."

Having enumerated the deficiencies and errors belonging to the chemistry of the vital processes, the author passes to the method and principles by which alone this science can be made to fulfil its just requirements. The final result of all physiologico-chemical investigations is assuredly to gain an accurate knowledge of the progress and connexion of the chemical phenomena attending the vital processes. It is needless to prove that we must thoroughly understand the substrata of the metamorphosis of the animal tissues before we can venture an opinion on the nature of the processes. The surest supports of physiological chemistry are to be sought, therefore, in general organic chemistry; while the study of the organic substrata of the animal body, must necessarily constitute an integral part of physiological chemistry, and prove a most efficient aid towards its development.

After having in this manner familiarized ourselves with the organic substrata of the animal body, we are still only on the threshold of the study of the constitution and functions of the animal juices and tissues.

"The study of the zoo-chemical processes based on zoo-chemistry and the theory of the animal juices, appertains to the third section of physiological chemistry, the theory of the metamorphosis of tissues—of nutrition and secretion. It has already been observed, that the actual object of physiological chemistry is to examine the course of the chemical phenomena of the animal organism in their causal connexion, and to deduce them from known physical and chemical laws; or in other words, to explain them scientifically. Even if we regard the chemical substratum, as made known to us by zoo-chemistry and the theory of the juices in the light of a satisfactorily investigated question, there are still several directions to be pursued before we can reach the proper object of our enquiries. It is here most essential that we should be well acquainted with the paths to be followed, for in our search after truth we are compelled to call to our aid hypotheses which might easily lead us into the domain of pure fiction.

"As long as zoo-chemistry and the theory of the juices continue to occupy their present subordinate position, the only method by which the foundation necessary to an exact investigation can be obtained, is that which we may term the statistical. Liebig, Boussingault, and Valetin have indeed, with a more correct view of what was required, attempted to compare the final effects of the whole with the material substrata supplied to the organism. We cannot, it is true, arrive at any conclusion regarding the working of the process itself by a mere juxtaposition and quantitative comparison of the ingesta and excreta of the animal organism, any more than we can judge of the causes and course of diseases by the number of fatal cases recorded; but such experiments furnish us with certain general results which serve as guides to further investigations. Some of the most important questions, whose solution was specially necessary, were unanswerable by any other method. Thus, for instance, it was ascertained, by an accurate investigation of the food, and its comparison with the constituents of the excreta and of the nutrient fluids, that in the ordinary food of animals, albuminous substances occur in sufficient quantity to compensate for the nitrogenous matters lost in the process of nutrition and in the metamorphosis of tissue; while it was thus at the same time shown, that the animal organism does not necessarily possess the property of generating albuminous matter from other substances containing nitrogen. The question whether the animal organism possessed the property of generating fat was answered in a similar manner; and it is well known that by means of such statistical observations, (comparing the fat contained in the food with that secreted in the cellular tissue and mixed with the excrements) the contest carried on between Liebeg on the one side, and Dumas and Boussingault on the other, regarding the formation of fat, was finally decided in favor of the former.

"This statistical method preserves us from setting up untenable hypotheses, and prosecuting useless experiments. How long were the minds of natural philosophers haunted with the illusion that animal bodies possessed the power of generating mineral elements, as lime, iron, sulphur, &c., from other elements, or even from nothing! It was this method alone which exposed the perfect nullity of the obstinately defended dogma of the 'vital force.'"

The author follows up this line of argument by allusion to other applications of the statistical method as one of the most important aids toward a solution of some of the more general questions in reference to the metamorphosis of the animal tissues. In addition he refers to the *comparative analytical* or *chemico-experimental* method, by which he may examine the cause of phenomena and the cause of their succession, in as far as the chemical phenomena of the living body may be artificially imitated, and the chemical metamorphoses of certain substances, external to the vital sphere, be compared with those within the influence of the vital processes. Liebig and his school have here done essential service.

"Liebig was led to believe from his statistical enquiries on fats, that these substances in their transmission through the organism, were in a great measure oxidised and reduced to water and carbonic acid, by which means they specially contributed towards the maintenance of animal heat. As he was by no means inclined to believe, as some have supposed, that fat was consumed in the lungs, somewhat in the same manner as oil burns in a lamp, it was necessary more accurately to investigate its gradual metamorphosis, and its transition through different stages of oxidation, and into bodies containing a larger quantity of oxygen. He believed that he could most readily attain this object by the comparative analytical method; and hence he and his school entered upon a series of experiments on the numerous products of decomposition of fatty matters, and more especially on their products of oxidation; and although we may still be far removed from the object in view, these enquiries have enriched us with many valuable results. A similar instance is afforded by the gelatigenous tissues of the animal body; for although our histological and statistico-chemical investigations leave not the slightest doubt that the gelatin is formed from the albuminous matters, the process of this metamorphosis is still wholly unexplained; and before we shall be justified in forming an opinion regarding this metamorphosis, and expressing it by a chemical equation, it is indispensably necessary that we should investigate the metamorphoses experienced by albuminous bodies during their gradual oxidation.

We are indebted for these views to the admirable investigations prosecuted under Liebig's direction, by Schlieper and Guckelberger, on the products of oxidation of albuminous bodies and of gelatin."

We have no wish to trespass on the patience of our readers by extending our quotations from Lehmann to an unreadable length, and we will therefore content ourselves by giving a few sentences from the concluding paragraph of his "Introduction."

"In conclusion, we would advance a few remarks on the place which physiological chemistry occupies, or at some future period will occupy, among the auxiliary medical sciences. If the final result of all physiologico-chemical enquiries be that of comprehending the chemical phenomena of animal life in their different phases and in their causal connexions, it is obvious that we must look to this science for a solution of the most important questions of physiology, and of medicine generally. It cannot be denied that most of the phenomena of animal life either consist in or are accompanied by chemical processes; nor can we form an adequate conception of the functions of the nervous system by which sensuous perception and motion are regulated, without the simultaneous existence of chemical actions. For although we are as yet unable to make nervous action fully harmonise with definite physiological laws, or to identify it with certain physical forces or imponderable fluids, all physiological experiments indicate that it is always followed by a chemical reaction, and that the nervous system experiences chemical changes by and through its own activity. It must, indeed, be admitted that any actual proof of such chemical metamorphoses is at present perfectly unattainable, and that our chemical methods would here afford us no higher aid than that which the scalpel yields to the pathological anatomist. But ought we to despair of attaiding our object, because we do not as yet clearly perceive the direction we are to follow? Weariness of the senses is the diminished impressibility of the nerves of sense, but its cause cannot reasonably be sought for in any other than a chemical change, experienced by the conducting substance of the nerves. Such a chemical metamorphosis of the nerves of sense from external impressions can no longer greatly excite our astonishment, since we have witnessed the unexpected phenomenon of a picture produced suddenly, and as it were by magic, from the chemical changes effected by the rays of light on an iodised silver plate. Should we not be equally justified in saying that the iodised plate, which after being exposed for a few seconds to a strong light gives only faint and half effaced images, is wearied like the retina, when after repeated and continuous perception of an image, it gives back only the faint outlines of the object? We may rest assured that the nervous system is not exempt from chemical action; and if the nervous system itself must fall within the domain of chemical contemplation, and a chemical expression remains to be found for its action, no less than for that of digestion and for the formation of blood, it is scarcely necessary to offer further proof of the fact that chemistry is destined to play the most important part in physiology and medicine. However much we may endeavor to exclude chemistry from certain physiological investigations, we shall always find that it involuntarily forces itself upon our notice; for without it we shall be unable to find a physiological equation or a philosophical expression for a process. In a scientific point of view chemistry must, therefore, be regarded as an invaluable acquisition to physiology. We have, then, little cause to dread that Cicero's observation "*Suo quisque studio delectatus alterum contemnit*," will be applied to ourselves when we assert that physiological chemistry is the crowning point of every physiological enquiry.

"When we turn to practical physiology, to pathology, and therapeutics, we are again reminded that chemistry is indispensable. Is there a single disease that is not attended by chemical changes? Can we ever hope to comprehend or explain the nature of any process, if we are ignorant of its integral factors? Life cannot exist without chemical movements, disease cannot exist without chemical changes. Thus much in reference to pathology; while in respect to therapeutics, it is almost superflous to observe that chemistry here also plays the principal part, for where has modern pharmacology sought its chief support, save in chemical processes and principles? And if we have advanced so far towards a clear insight as no longer to ascribe supernatural forces to medicines, but to derive their efficiency especially from chemical properties, then must chemistry be the supporting basis of pharmacology. The physician acts upon the body mostly by the aid of matter, which retains its characteristic powers within no less than without the organism. If then nervous action likewise falls within the sphere of chemical metamorphoses, the *Nervina* (or *Neurotica*) of pharmacologists must primarily at least act chemically on this system."

We are reminded by this argument of our author, of a remarkable passage in one of the lectures of Dr. John Simon, F. R. S., of London, on general pathology. He is speaking of the use of excretive drugs in relation to humoral pathology, (lecture xi.,) and after announcing that "within the compass of pathology, I know of nothing so obscure and unsatisfactory as that division of the science which relates to morbid and medicinal changes in the excretory functions:" he proceeds to contrast the vagueness and uncertainty of our knowledge of the action of remedial agents with what occurs in chemistry and physics—and discourses in the following remarkable and suggestive manner:

"We are led to an intimate pharmaceutical acquaintance with the *Materia Medica.* We learn the smell of rhubarb, the tests of arsenic, the chemical incompatibilities of iron, the adulterations of quinine or sarsaparilla, and so forth. We become skilful dispensers. We know that bark very often cures ague; that copaiba frequently stays a clap; that cod-liver oil and iodide of potassium between them relieve a good many cases of scrofula; that taraxacum is sometimes useful below the diaphragm, and squill above it. And with this farrago of traditions, the misnamed *science of Materia Medica* has remained so contented and so stationary, that at the present moment—in the middle of the nineteenth century—we do not possess a complete medical knowledge of any single article of the Pharmacopœia.

"It would be easy for one quite ignorant of medicine to predict, on general grounds, what must be the result of this state of things; what blind reliance on specifics where they are supposed to prevail; what credulous—perhaps rather what desperate combinations of drugs where we have no traditional specific to trust to. An indifferent shot in a turnip-field does not mark his one bird and bring it down; he bangs at the whole covey, and thinks it a hard case if he hits nothing. On some such principle as this—scarcely sanctioned in the sportsman, much less admissible in the medical philosopher—we too often discharge our barrels. If the immemorable *certificates* of a specific serve us instead of real knowledge, and enable us confidently to give quinine to one patient, and sulphur-ointment to another—good; but in the absence of such traditions, how many prescriptions are written, which combine at least a dozen different materials, where the prescriber is utterly unable to define the precise errand on which any one of these ingredients is sent into his patient's body. This prescription failing another of the same sort is written, and another after that; and presently the patient dies or recovers, as the case may be. And in either event, from the absence of a definite principle in the treatment, and from the want of having attached a separate meaning and intention to each drug employed, the pharmaceutical experiment has been too complex for the practitioner to draw any rational or useful inference as to the immediate causes of his success or failure.

"Contrast this with what occurs in chemistry or in physics. You wish to precipitate a certain material from its solution; you do not throw in at random the contents of several vials, with a vague hope that one of these re-agents may answer your purpose; you proceed with a very definite object, and by appropriate means.

If A be present (you say) B will throw it down, by forming such or such insoluble combination—provided (you may perhaps have to add) that C be not present likewise, for in that case C must, first of all, be neutralized; provided also, (you may further have to concede) that B be added in a sufficient, and not in an excessive quantity, lest it re-dissolve its own precipitate. These, or similar allowances being made, the sources of fallacy are just as definite and palpable as the anticipated positive result; and, if your operation should miscarry, you would be able to account for its failure, and to repeat it in a form which—not casually, but certainly—would accomplish your purpose. So, again, if you plunge the wires of your galvanic battery into water, and fail to produce the expected result, you do not fly off to some new arrangement—you do not try wooden conductors, or add twelve additional wires, or attempt to decompose the fluid with your finger and thumb; you know exactly what the hitch must be, that your circuit must be incomplete, or that you have forgotten the acid, or something of this kind; you correct your omission, and you are certain of the result—*certain*, because you know definitely the means to attain it, and are cognizant of their method and principle of operation. On the other hand, when we have given colchicum to six patients suffering with gout, when it has relieved two of them, and has left four unrelieved, we are quite unable to explain why it failed here, and succeeded there; and consequently, in that unsuccessful majority, we are quite unable to supply the absent condition (whatever it may have been) which was accidentally present in the successful instances, and conduced essentially to their cure.

"It is quite indispensable for the progress of medicine—I might say, indispensable for its existence as a science—that our *Materia Medica* should be made subject to a true pharmacology; that its province should cease to be a mere emporium of recipes; that we should have a knowledge of its various elements in their true relation to the living body; that we should give drugs only with a clear perception of their causativeness, and with a definite object before us; understanding the medicine as well as the malady, and taking one good aim at the substance of the disease, instead of discharging a volley at its shadow."

After showing the close and inseperable union which must subsist between pathological and physiological chemistry—and the absurdity of rejecting the evidence of the former on the ground that it deals with abnormal conditions—our author lastly enters a warm protest against the transcendental doctrines of what is called metaphysiology. The recent advances of organic chemistry have unfortunately been interwoven with a fantastic physiology, which designates itself as a comparative science. Comparing together the *functions* of the organs of different animals as comparative anatomy compares their structure, is not a science, but a system founded on abstractions and ideal comparisons. We entertain all due respect for that form of metaphysics which occupies the same rank among the speculative sciences which chemistry and physiology hold among the exact sciences. But metaphysics and physiology may be likened to two divergent lines, which coincide only in their starting point, never again to unite. "We therefore believe with the diffidence beseeming true students of nature, that it would be wiser, and more conducive to the spread of true knowledge, to adhere in the study of vital processes, to matter and to the laws by which it is determined, than, following the fictitious abstractions of dynamical processes to assume that there exists in life a higher power of the spiritual force pervading matter.

"We are firmly convinced that even metaphysiology will be unable to deprive physiological chemistry of the consideration due to it among physical studies, in its explanation of vital processes; and we will, therefore, leave it to the poetic and the imaginative to depict the romance of the protecting activity and sturdy contest maintained by the vital force, and of a struggle between different powers,—between the attraction and repulsion of polarities. Does it not need a superabundant richness of fancy to believe with metaphysiologists, that apparent death, trance, or (as it has been termed) latent life, is the predominance of the spiritual over the material (the metamorphosis of matter being at its minimum) rather than a predominance of the material over the spiritual, as sounder minds would be led to assume? It would be well if these spiritualists would look down from the high stand they have chosen, and deign to believe that there are some among those experimentalists, who, clinging to matter, and gathering their facts with ant-like industry from the lowly earth, notwithstanding that they have long held communion with the poet-philosopher, Plato, and the philosophical natural inquirer, Aristotle, and have some familiarity with the Paraphrases of Hegel and Schelling, are yet unwilling to relinquish their less elevated position. If these happy admirers of their own Ideal had descended from their airy heights and closely examined organic matter, they would not have deemed it necessary to assume, that besides carbon, hydrogen, nitrogen, and oxygen, organic substances must also contain an *organogenium* or latent vital force, or whatever else they may be pleased to call it. Had they sought information from a chemist, they would have learnt, that when exposed to the clear light of rigid logic, there is no essential difference between organic and inorganic bodies; a chemist totally unacquainted with organic matter, would *a priori* have deduced all these incidental differences of matter, from the doctrine of affinity and the science of stoichiometry, evolved from dead matter. However these advocates of a romantic poetry of nature may despise the swarm of industrious investigators, who are often unwearyingly occupied for years together in endeavoring to collect a few firm supports for the great edifice of a true philosophy of nature, we do not despair of seeing our work rise in simple grandeur, more durable and lasting than those sophisms of natural philosophy which, passing through ages from Pythagoras and Empedocles to Schelling and Hegel, have, like the sand of the ocean shore, been alternately upborne by one wave and engulphed by the next."

Having thus followed our author to the threshold of his subject we must bring our article to a close and wait the publication of his final volume on the transformation of the tissues and the great vital processes, satisfied if we have been able to indicate clearly the line of his argument and the spirit of safe, cautious and practical philosophy which animates his work. We trust that an American reprint of LEHMANN's Chemistry will be undertaken in order that the work, now comparatively inaccessible and very costly, may be brought within the reach of all students.

---

ROBIN AND VERDEIL (*Chimie Anatomique*) have produced a work of great labor, covering much the same ground as LEHMANN. It is elaborated in the best style of the French school, and in respect to its thoroughness of bibliographical and literary research leaves nothing to be desired. It is entirely out of the question that we should enter at all on its merits at present. The authors are well known as among the most laborious, earnest workers in "young France." This present treatise is accompanied by an atlas of microscopic plates of the crystalline bodies found in the fluids and cavities of the animal organism, drawn and colored from nature, by the authors, which surpasses everything ever done before in the same line, not even excepting the faithful excellence of Dr. FUNKE.

The discovery by VERDEIL of a new and peculiar acid (the *pneumic*) in the substance of the lungs has led to the broaching of a novel theory or explanation, for the origin of the carbonic acid of respiration, as being the product of the action of this acid upon the alkaline bicarbonatis of the blood. This new view, so much at variance with the "combustion theory," is discussed at some length, and with much ingenuity. Taking the facts as they are stated, it shows how far we yet may be from having conquered the first foot of stable ground, in the still new domain of this most vital function.

